# Novel Reporter System Monitoring IL-18 Specific Signaling Can Be Applied to High-Throughput Screening

**DOI:** 10.3390/md18010060

**Published:** 2020-01-17

**Authors:** Riho Kurata, Kenji Shimizu, Xiaofeng Cui, Masamitsu Harada, Takayuki Isagawa, Hiroaki Semba, Jun Ishihara, Koji Yamada, Jun Nagai, Yasuhiro Yoshida, Norihiko Takeda, Koji Maemura, Tomo Yonezawa

**Affiliations:** 1 Education and Research Center for Pharmaceutical Sciences, Osaka University of Pharmaceutical Sciences, 4-20-1 Nasahara, Takatsuki, Osaka 569-1094, Japan; kurata@gly.oups.ac.jp; 2 Division of Immune Regulation, Institute for Genome Research, Tokushima University, Tokushima-shi, Tokushima 770-8505, Japan; kshimizu@genome.tokushima-u.ac.jp; 3 School of Chemistry, Chemical Engineering and Life Sciences, School of Materials and Engineering, Wuhan University of Technology, 122 Loushi Rd, Wuhan 430070, Hubei, China; xfc.cui@gmail.com; 4 Center for Therapeutic Innovation, Gene Research Center for Frontiers Life Sciences, Nagasaki University, Graduate School of Biomedical Sciences, 1-12-14 Sakamoto, Nagasaki 852-8523-0022, Japan; vokuaoto@me.com (M.H.); jnagai@partners.org (J.N.); 5 Department of Cardiovascular Medicine, Nagasaki University Graduate School of Biomedical Sciences, 1-7-1 Sakamoto, Nagasaki 852-8501, Japan; i-takayuki13@nagasaki-u.ac.jp (T.I.); maemura@nagasaki-u.ac.jp (K.M.); 6 Department of Cardiovascular Medicine, The Cardiovascular Institute, Tokyo Japan Nishiazabu, 3-2-19, Minato-ku, Tokyo 106-0031, Japan; hiroaki_se@yahoo.co.jp; 7 Graduate School of Biomedical Sciences, Nagasaki University, 1-14 Bunkyo-machi, Nagasaki 852-8521, Japan; jishi@nagasaki-u.ac.jp (J.I.); kyamada@nagasaki-u.ac.jp (K.Y.); 8 Department of Immunology and Parasitology, University of Occupational and Environmental Health, 1-1 Iseigaoka, Yahatanishi-ku, Kitakyushu 807-8555, Japan; freude@med.uoeh-u.ac.jp; 9 Department of Cardiovascular Medicine, Graduate School of Medicine, The University of Tokyo, 7-3-1, Hongo, Tokyo, Bunkyo-ku 113-8654, Japan; ntakeda-tky@umin.ac.jp

**Keywords:** EBNA1, pNFkB-SEAP, IL-18Rap, IL-18R1, drug screening, marine bacteria, synthetic compound

## Abstract

Very recently, the immunotherapies against cancer, autoimmune diseases, and infection have been feasible and promising. Thus, we have examined the possibility whether or not human gamma delta T cells can be applied for the novel immunotherapies. We previously established the cells stably maintaining NFkB-driven human secreted embryonic alkaline phosphatase (SEAP) expression. The cells can be used to determine the transcription activity of NFkB with high-standard dynamic range and accuracy. Because IL-18 is a kind of cytokines that enhances cytotoxicity and activity of human gamma delta T cells through NFkB activation, we have focused on the activity and signaling of IL-18. In this study, we modified the previous reporter cell that can determine the transcription activity of NFkB to express two subunits consisted of human IL-18 receptor. The modified cells secreted SEAP in response to treatment with human recombinant IL-18 in a concentration-dependent manner. We also observed the concentration-dependently enhancement of NFkB activity in the cells treated with mouse recombinant IL-18 although the affinity was lower compared to human recombinant IL-18. We also previously established the cells stably expressing and secreting human recombinant IL-18 and then validated whether or not the conditioned medium from the cells activate NFkB transcription activity using this assay. Our university has kept collecting many extracts from over 18,000 marine bacteria in our local sea around Omura bay—fungi, plants for Chinese herbal medicine, and so on—and also have kept gathering synthetic compounds from many Japanese chemists as drug libraries. Finally, in order to identify drugs mimicking IL-18 biological activity or possessing inhibitory effects on IL-18-induced NFkB, we demonstrated drug screening using number of extracts derived from marine bacteria and synthetic compounds.

## 1. Introduction

The immunotherapies against cancer, autoimmune diseases, infections, and so on have developed eminently [1]. Very recently, PD-1 antibody-induced immune checkpoint blockade [2] using Nivolumab or chimeric antigen receptor-T cells (CAR-T), which is a Tisagenlecleucel also well known as Kymriah [3], have been shown to have feasible therapeutic effects on cancer development. We have validated whether or not human gamma delta T cells can be used for novel immunotherapy and previously demonstrated conditioned medium from cells stably secreting human recombinant Interleukin (IL)-18 activates human gamma delta T cells [4]. IL-18 is one of pro-inflammatory cytokines activating both innate and acquired immunities [5]. It was firstly recognized as Interferon gamma (IFNγ)-inducing factor (IGIF) [6]. In liver of mouse infected with *Proprionibacterium acnes*, Kupffer cells, and macrophage-enhanced IFNγ production from T and Natural Killer cells through a production of IGIF in secondary challenged with an intraperitoneal endotoxin [6]. After cloning the gene of IGIF, renamed it to IL-18 because the molecule has similar structure of IL-1 family [7]. At first, IL-18 is also translated as an inactive precursor with no signal peptide and then, proteolytically cleaved by caspase-1 to become an active form [8]. 

Cognate receptors consist of IL-18 receptor alpha chain (IL-18Ra), which has lower affinity but binds to mature IL-18, and beta chain (IL-18Rb, a.k.a. IL-18R1), which can be formed with a higher affinity complex to mature IL-18 [9,10]. IL-18 receptors have Toll-IL-1 receptor (TIR) domains and recruit myeloid differentiation primary response protein (MyD) 88, four IL-1 receptor associate kinases (IRAKs), and tumor necrosis factor receptor associated factor (TRAF)-6, resulting in an activation of nuclear factor-kappa B (NFkB) [11]. The IL-18Ra is ubiquitously expressed in the most cells, while IL-18Rb is restricted in immune cells such as dendritic cells and T cells including gamma delta T cells [11].

Regarding agonists and antagonists, GSK1070806 is established as a humanized IgG1antiboy, which can bind to human IL-18 with a high affinity, and neutralizes its function—although there is a natural neutralizing binding protein (IL-18BP) [12,13]. While clinical grade human IL-18 therapy (SB-485232) was used in clinical trials [14,15,16,17]. However, small molecules modulating IL-18 effects have not yet been identified.

In this study, in order to establish a monitoring system specifically for IL-18-induced NFkB activation, we constructed expression cassette consisting of hIL-18Ra and b combined with P2A peptide, which is a kind of self-cleaving peptide derived from a type of foot-and-mouth virus [18,19]. Besides that, the expression cassette was applied to use Epstein–Barr virus nuclear antigen (EBNA) 1 and origin of plasmid replication (OriP) as well as we previously established NFkB, interferon regulatory factor (IRF)-reporter cells [14] or stably expressing and secreting active recombinant hIL-18 [4]. We introduced it to the previously established cells stably NFkB-driven SEAP expression. Then, we succeeded in monitoring IL-18 specific NFkB activation in response to recombinant human or mouse IL-18 although their affinities are different. We also validated whether or not it can be applied to high-throughput screening (HTS) and thus, it has high-dynamic range and accuracy to carry out it. Finally, we demonstrated several HTS using natural extract library derived mainly from marine bacteria and synthetic compound library and then, we identified several extracts and compounds with inhibitory effects on IL-18-induced NFkB activation.

## 2. Results

### 2.1. Design of Construct Stably and Simultaneously Expressing Two Cognate Human IL-18 Receptor Subunits

**Figure 1:**
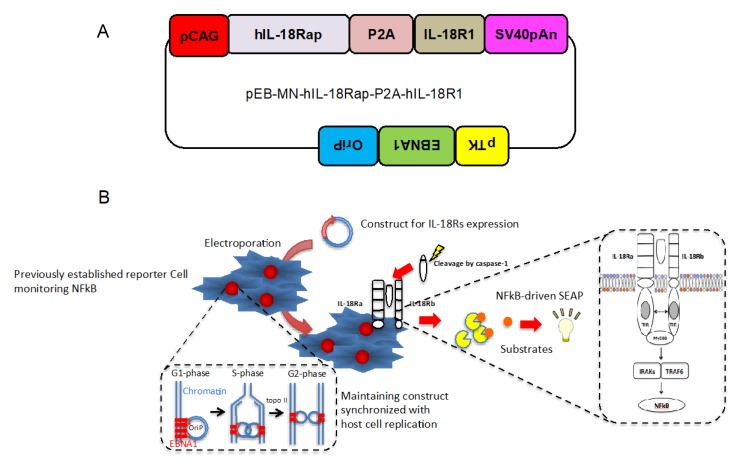
Design of construct stably and simultaneously expressing two cognate human IL-18 receptor subunits. (**A**) The structures of a novel constructs, pEB-MN-hIL-18Rap-P2A-hIL-18R1. (**B**) Schematic figure indicates procedures of electroporation to introduce the construct into the previously established NFkB reporter cells and maintaining the constructs synchronized with host cell replication and stably expressing both human IL-18R accessory protein and IL-18 receptor 1 in order to detect specifically IL-18-induced NFkB.

In order to obtain stable and simultaneous expressions of human IL-18Ra and b in the NFkB reporter cells, we designed and established a novel construct that includes combined human IL-18Ra to b by P2A peptide and then, introduced to EBNA1 and OriP backborne as shown in Figure 1A. Next, we introduced the vector or empty vector (pEB-Multi-Puro) to the NFkB reporter cells using electrophoresis as shown in Figure 1B. After that, we selected the cells with puromycin and G418. We expected the cells maintain both NFkB reporter and expression of human IL-18 receptor subunits synchronized host cell replication (Figure 1B).

### 2.2. Establishment of Cells Specifically Monitor IL-18- Induced NFkB Activation

**Figure 2:**
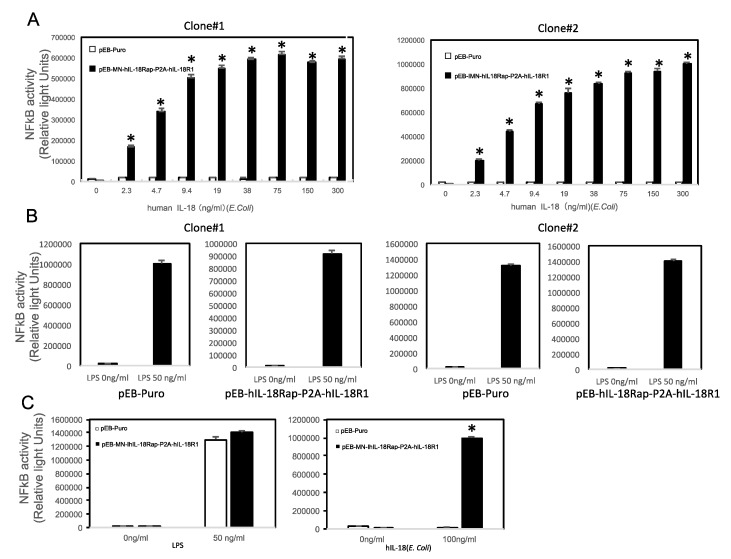
Establishment of cells specifically monitor IL-18- induced NFkB activation. (**A**) Human recombinant IL-18 activates NFkB in the cells introduced with pEB-MN-hIL-18Rap-P2A-hIL-18R1 (closed bar) in a concentration-dependent manner, but did not in the cells introduced with empty vector (open bar). (**B**) LPS activates NFkB both in the cells introduced with pEB-MS-hIL-18Rap-P2A-hIL-18R1 (right) or empty vector (left). (**C**) Human recombinant IL-18 (right part) activates NFkB only in the cells introduced with pEB-MN-hIL-18Rap-P2A-hIL-18R1 (closed bar) but not empty vector (open bar) although LPS (left part) activates in both the cells. Two independent clones were validated (left part: clone#1 and right part: clone#2). The data are expressed as means ± SEM (*n* = 3 or 4). * was considered statistically significant if their *P* values were 0.05 > *P*.

In order to examine whether or not NFkB activation specifically can be occurred in the cells in response to IL-18, we treated various concentration of human recombinant IL18 with the cells introduced the construct or empty vector. In both two independent cell clones, we could observe IL-18-induced NFkB activation in a concentration dependent manner; however, in the cells introduced with the empty vector, it could not be observed (Figure 2A). We also observed LPS-induced NFkB in both of the clones with or without the construct (Figure 2B and C left). We emphasized human IL-18-induced NFkB activation could be observed in the cells introduced with the construct expressing human IL-18 receptor subunits (Figure 2C right). 

### 2.3. Human and Mouse IL-18-Induced NFkB Activation and Their Affinities

**Figure 3:**
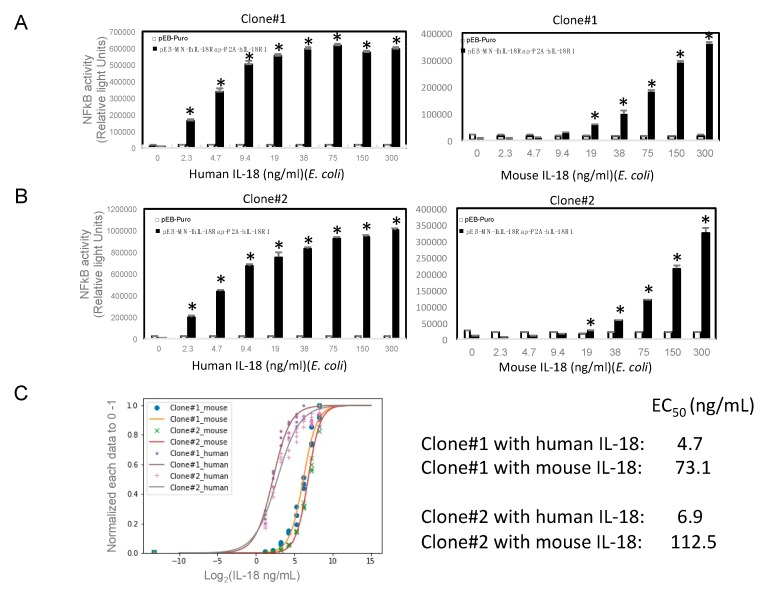
Human and mouse IL-18-induced NFkB activation and their affinities. Human (left part) or mouse (right part) induces NFkB activation in a concentration-dependent manner in the clone #1 (**A**) or #2 (**B**). Closed and open bars indicate the cells introduced with pEB-MN-hIL-18Rap-P2A-hIL-18R1 and empty vector, respectively. EC_50_ values were calculated by Logistic curve fit and concentration-dependent data (**C**). The data are expressed as means ± SEM (*n* = 3 or 4). * was considered statistically significant if their *P* values were 0.05 > *P*.

We next tried to examine whether or not there are differences of responsiveness between human and mouse recombinant IL-18 in the cells. We treated various concentrations of human or mouse recombinant IL-18 ranging from 2.3 to 300 ng/mL to the cells and then, determined NFkB activity. We could observe the concentration-dependency of human (Figure 3A and B left) and mouse (Figure 3A and B right) IL-18-induced NFkB activation. Any activation of NFkB could not be occurred in the parental cells treated with human or mouse IL-18 (Figure 3A,B). However, to a lesser extent, mouse IL-18 activates NFkB in the novel reporter cells (Figure 3A and B right). Thus, we tried to calculate half maximal of effectiveness in mouse or human IL-18 treatment. Our curve fit analysis indicated that EC_50_ of human IL-18 in two independent experiments were 4.7 and 6.9 ng/mL, while those of mouse were 73.1 and 112.5 ng/mL (Figure 3C).

### 2.4. Novel Reporter Assay can Validate IL-18-Specific NFkB Activity in Supernatant

**Figure 4:**
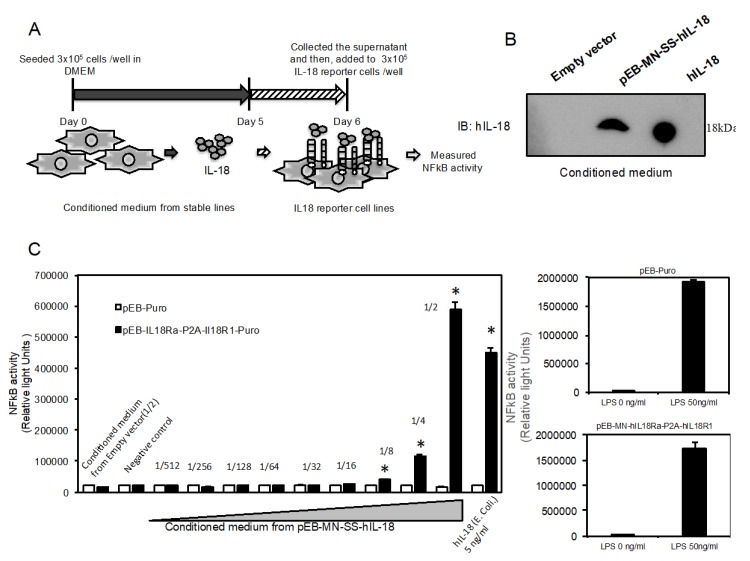
Novel reporter assay can validate IL-18-specific NFkB activity in supernatant. (**A**) Schematic figure indicates procedures for preparing each conditioned medium from the previously established cells stably secreting human recombinant IL-18 proteins and then, treatment of the conditioned medium with the novel reporter cells. (**B**) Validation of human IL-18 protein expression in the conditioned medium using western blotting. Human recombinant IL-18 protein generated from *E. coli* was used as positive control (1.5 ng). (**C**) Validation of the supernatant serially diluted from 1/2 to 1/512 in the novel reporter cells (closed bar) or the cells introduced with empty vector (open bar). Then, LPS induces NFkB activation in both of the two cells (right part). The data are expressed as means ± SEM (*n* = 3 or 4). * was considered statistically significant if their *P* values were 0.05 > *P*.

In order to examine whether or not the reporter cells can validate IL-18 activity in supernatant, we prepared supernatant from the cells stably expressing and secreting active human IL-18 protein as shown in Figure 4A. We could check substantial amount of human IL-18 proteins by immnoblotting (Figure 4B). Then, we serially diluted to various concentration by adding to culture medium in the reporter cells. Twenty-four hours after that, we measured SEAP activity in the supernatant. The dilution from 1/2 to 1/8 of the conditioned medium significantly activates NFkB activity in the reporter cells (Figure 4C left), while in the parental cells introduced with empty vector, any activation of NFkB was not observed (Figure 4C left). LPS activates NFkB activity in both of the two cells (Figure 4C right).

### 2.5. High-Throughput Screening in the Novel Reporter Cells Using Natural Product and Synthetic Compound Libraries

**Figure 5:**
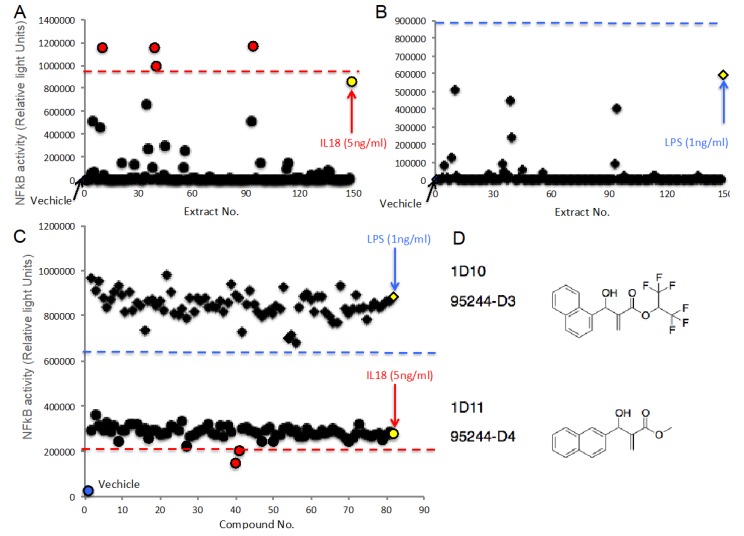
High-throughput screening in the novel reporter cells using natural product and synthetic compound libraries. The result of HTS in the novel reporter cells (Circle) (**A**) or the cells introduced with empty vector (Rhombus) (**B**) treated with 147 extracts derived from mainly marine bacteria (**A**). Yellow circle and rhombus indicate ligands such as 1 ng/mL IL-18- or 1 ng/mL LPS-induced NFkB activities, respectively (*n* = 8). Blue circle and rhombus indicate vehicle (0.5% DMSO). Dashed line indicates threshold as average of positive control, such as IL-18 (red) or LPS (blue), +6SD and then, identified four extracts (red circle). (**C**) The result of HTS in the novel reporter cells (red and lower part) or the parental cells introduced with empty vector (blue and upper part) treated with 80 of 240 synthetic compounds provided by Japanese chemists in the presence of 5 ng/mL IL-18- or 1 ng/mL LPS. Yellow circle and rhombus indicate only ligands such as 5 ng/mL IL-18- or 1 ng/mL LPS-induced NFkB activities, respectively (*n* = 8). Blue circle and rhombus indicate vehicle (0.5% DMSO). Dashed line indicates threshold as average of positive control, such as IL-18 (red) or LPS (blue), +6SD and identified two compounds (red circle). (**D**) Structures of identified two compounds.

Finally, we tried to perform HTS using natural product library, which is mainly extracted from marine bacteria, and synthetic compound library, which is provided by Japanese chemists. As shown in Table 1, S/B and S/N, which are important in dynamic-range and assay quality, and Z’ factor, which is a measure of the statistical characteristic for HTS, were fulfilled for general criterion to carry it out. We could obtain high-standard dynamic range and high-accuracy assay in independent measurements even if half maximal concentration of human IL-18 (Table 1). To identify agonistic matters mimicking human IL-18, the cells were incubated with 147 extracts derived from marine bacteria, which were diluted at 1/200, or 240 synthetic compounds at 1 µM for 24 h and then, NFkB activities in the supernatant were determined. We could get high-quality signal compared to vehicle in both extracts from marine bacteria (Figure 5A) and synthetic compounds (Supplementary Figures). We set the threshold as average of positive control +6SD and then, identified four extracts as ‘Hit’ (Figure 5A). The Z’ factor was 0.737. While, we could not identify any hit among synthetic compounds (Supplementary Information). We also performed HTS in the parental cells introduced with empty vector as counterscreen assay and then, could not observe any hits (Figure 5B). The Z’ factor was 0.556. To identify matters possessing inhibitory effect on IL-18-induced NFkB, the cells were incubated with 147 extracts derived from marine bacteria, which were diluted at 1/200, or 240 synthetic compounds at 1 µM in the presence of human IL-18 for 24 h and then, NFkB activities in the supernatant were determined. The threshold was assigned as average of positive control—6SD and then two compounds were identified (Figure 5C lower). In counterscreen assay using LPS, we could not observe any hit (Figure 5C upper). The Z’ factors of these were 0.911 and 0.80, respectively. The two hit compounds had similar structures (Figure 5D). 

## 3. Discussion

In this study, for the first time, we established novel reporter cells stably expressing human IL-18 receptor subunits, which are consisted of human IL-18Rap and IL-18R1, by modification of the previous established reporter cells [14]. In the reporter cells, we succeeded in detecting specifically IL-18-induced NFkB activation in response to human recombinant IL-18 protein. The assay, which has high-dynamic range and high-accuracy, can be used to HTS even if low concentration of human IL-18 or LPS in novel or our previous reporter system, respectively [20]. This assay system could validate biological active IL-18 protein in the culture medium from the cells secreting it. It is useful to do biological activity of IL-18 as well as KG-1 cell line, which is a human acute leukemia and a standard to validate it by secreting IFN-γ in culture media [21]. Mouse recombinant IL-18 also induced NFkB activation although its grade is lower than human. Indeed, our curve fit analysis indicated that the responsiveness of mouse IL-18 was lower than that of human by comparison of each half maximal concentration. Besides that, homology between human and mouse IL-18 is only 64.5% in amino acids. Moreover, those of IL-18Rap and IL-18R1 are also only 66.9% and 64.4%, respectively (data not shown). In our preliminary experiment, we prepared conditioned medium from cells expressing mouse IL-18 active form as well as human and then, validated NFkB activation by similar serial dilution. However, we failed to detect any significant activation of NFkB. Taken together, we concluded that heterogeneous IL-18 must have lower affinity to counterpart of IL-18 receptors according to their homologies. 

Eventually, we set out to perform HTS using extracts from marine bacteria and synthetic compounds in order to identify agonist or antagonist of IL-18-induced NFkB signal. Nagasaki University and Prof. Yamada’s group have kept collecting many extracts from over 18,000 marine bacteria in our local sea around Omura bay—fungi, plants for Chinese herbal medicine, and so on—and prepared their extracts as a drug library. Prof. Hatakeyama and Prof. Ishihara’s group has kept gathering synthetic compounds including a novel structure provided from many Japanese chemists at Universities or Institutes and provided these as a drug library. In this study, we used a part of these, which are 147 extracts and 240 compounds, and performed HTS. We succeeded in identifying four extracts mimicking IL-18-induced NFkB activity, while our counterscreen assay by LPS did not identify any extracts. It was first time for us to use these two libraries to perform HTS. These would be benefits for scientists to perform HTS. In future, we are going to fractionate the extract and then, identify a single ingredient affecting it. We also identified two compounds possessing inhibitory effects on human IL-18-induced NFkB activation. A counterscreen assay did not identify any compounds having an inhibitory effect on LPS-induced NFkB activation. Because LPS- or human IL-18-induced NFkB activation had their different extents in this assay, we could perform both the assay and the counterscreen assay in the same measurement. Strikingly, two hit compounds have a similar structure and are derivatives. These were synthesized using β-Isocupreidine-hexafluoroisopropyl acrylate method for asymmetric Baylis-Hillman reactions by Prof. Hatakeyama and Prof. Ishihara’s and their colleagues [22]. In the future, we are going to validate much more derivatives and examine its structure-activity relationship. To our knowledge, it was first time to identify small molecules inhibiting IL-18 activities.

In conclusion, we for the first time established the novel reporter cell line stably expressing human IL-18 receptor subunits and demonstrated the cells monitor IL-18 specific NFkB activation. It is a helpful tool to validate bioactivity human IL-18 in the novel reporter cell as well as KG-1 cells. Eventually, we performed HTS in order to identify agonist or antagonist in IL-18-induced NFkB signaling and then, identified four extracts and two compounds from extract and synthetic compound libraries.

## 4. Materials and Methods

### 4.1. Materials

PrimeStar GXL polymerase, Great EscAPe SEAP Chemiluminescent kit and DNA ligation kit, Mighty Mix was obtained from TaKaRa Bio, Shiga, Japan. pEB-Multi-Puro, skim milk and G418 were obtained from Wako Pure Chemical Industries, Ltd., Chuo-ku, Osaka, Japan. Four primers as shown in Supplement Information were obtained from Integrated DNA Technologies, Inc., Skokie, Illinois, IL, USA. Puromycin and fetal bovine serum (FBS) were obtained from Sigma Aldrich, St. Louis, MO, USA. Anti-human IL-18 polyclonal antibody (#PM014) and anti-GAPDH monoclonal antibody (#M171-3), and recombinant mature form human (#B001-5) and mouse (#B002-5) IL-18 (generated from *E. coli*) were obtained from MBL CO., LTD., Nagoya, Japan. ECL prime Western Blotting Detection System were obtained from GE Healthcare, Uppsala, Sweden. Extracts from marine bacteria and synthetic compound library were kindly gifted from Prof. Yamada and Prof. Ishihara, respectively. Complementary DNA (cDNA) derived from human peripheral blood mononuclear cells (PBMCs) was kindly provided by Dr. Isagawa.

### 4.2. Plasmid Construction

We cloned human IL-18Rap (NM_003853.3) open leading frame attached with SalI site and partially overlapping P2A using the following primer set. The sense primer: 5′-AAGTCGACATGCTCTGTTTGGGCTGGATATTTCTTTGGCTTGTT-3′ and the antisense primer: 5′-AGGTCCAGGGTTCTCCTCCACGTCTCCAGCCTGCTTCAGCAGGCTGAAGTTAGTAGCTCCGCTTCCCCATTCCTTAGGCTGGGAGCTCCTC-3′. We also cloned human IL-18R1 (AY192162.1) open leading frame attached with NotI site and partially overlapping P2A using the following primer set. The sense primer: 5′-GGAAGCGGAGCTACTAACTTCAGCCTGCTGAAGCAGGCTGGAGACGTGGAGGAGAACCCTGGACCTATGAATTGTAGAGAATTACCCTTGACCCTTTGGGTG-3′ and the antisense primer: 5′-TTGCGGCCGCTCAGTGGAGCCCTGAGCTTGTTTTCCTG-3′. Details are shown in Supplementary Information. PCR reactions were performed using high-fidelity enzyme, PrimeStar GXL polymerase and cDNA derived from human PBMCs. After first PCR amplification, two obtained cassettes were mixed at same amount 0.5 μL and then, added with other PCR reagents using the sense primer of human IL-18Rap and the antisense primer of human IL-18R1 as shown in Supplemental Information. The condition was run for forty cycles using the following protocol: 10 s denaturation at 98 °C; 15 s annealing at 55 °C; 3 min amplification at 68 °C. The PCR products were digested with SalI and NotI restriction enzyme, and inserted into the corresponding sites of the pEB-Multi-Puro. It was sequenced to confirm each sequence. After inserting it into the vector, digested with SalI and NotI restriction enzyme, and inserted into the corresponding sites of the vector. Eventually, we established a construct shown in Figure 1A.

### 4.3. Cell Culture and Electroporation

The cells as previously established [4,14], which are stably expressing and secreting SEAP and human IL-18, were cultured in DMEM (Invitrogen, CA, USA) containing 10% FBS at 37 °C with 5% CO_2_. Then, we introduced pEB-MN-hIL-18Rap-P2A-hIL-18R1 (10 µg) into 1 × 10^6^ cells using NEPA21 Transfection Electroporation (NEPA Gene Co., Ltd., Chiba, Japan) as described previously [4,14]. In brief, the condition is 115V, pulse width: 2.5 ms (second), pulse interval: 50 ms, times: 2 and attenuation rate: 10% in poring pulse, and 20 V, pulse width: 50 ms, times: 5 and attenuation rate: 40% in transfer pulse. After that, the cells were cultured in DMEM containing 10% fetal bovine serum, puromycin (250 ng/mL) and G418 (25 µg/mL). To prepare conditioned culture medium from the cells stably secreting human recombinant IL-18 as previously reported [4], we seeded 3 × 10^5^ cells onto 6-well plate in DMEM containing 10% FBS for 4 days and collected. The supernatant was serially diluted from 1/2 to 1/512 and then, incubated with the established reporter cells in this study.

### 4.4. SEAP Assay with Chemilluminascence

The established reporter cells were seeded at 3 × 10^3^ cells/well cultured onto 384 well plate, respectively. Twenty-four hours after the seeding, we treated with or without each concentration of human or mouse recombinant IL-18 (MBL CO., LTD., Nagoya, Japan) ranging from 2.3 to 300 ng/mL. We also treated with 147 natural extracts, which were diluted at 1/200, or 240 synthetic compound at 1 µM in DMSO (the final concentration is 0.5%) with or without LPS or human IL-18 for HTS. Twenty-four hours after the transfection, we collected the supernatant from the cells. SEAP activity in the supernatant was determined by Great EscAPe SEAP Chemiluminescent kit 2.0 (TaKaRa Bio, Shiga, Japan) according to manufacturer’s instruction. In brief, we added 4 volumes diluent buffer to supernatant and then, incubated at 65 degrees for 30 min in 384-well plate. After that, add 5 volumes SEAP substrate solution and then, kept at room temperature for at least 10 min. Finally, we measured chemilminescence intensity by PHERAstar FS (BMG LABTECH JAPAN L.t.d., Saitama, Japna). Nagasaki University and Prof. Yamada’s group have kept collecting many extracts from over 18,000 marine bacteria in our local sea around Omura bay—fungi, plants for Chinese herbal medicine, and so on—and prepared their extracts as a drug library. Prof. Hatakeyama and Prof. Ishihara’s group has continued gathering synthetic compounds, including a novel structure provided from many Japanese chemists at Universities or Institutes and provided these as a drug library. 

### 4.5. Western Blotting

We used antibodies, anti-human IL-18 polyclonal antibody (#PM014) to detect IL-18 in the supernatant. Equal amount of culture supernatants were subjected to 15% SDS-PAGE before being transferred onto polyvinylidene difluoride (PVDF) membranes. The membranes were blocked with 10% or 7.5% skim milk in PBS at room temperature for 1 h. The membranes were exposed to each primary antibody in 1% skim milk in phosphate buffered saline (PBS) at room temperature for 1 h, and subsequently, were exposed to HRP-conjugated secondary antibody at room temperature for 1 h. The membranes were then washed six times with PBS containing 0.05% Tween-20 (PBST), followed by an hour incubation at room temperature with secondary antibody, the membranes were then washed six times with PBST and were visualized using ECL prime Western Blotting Detection System and ChemiDoc^TM^ imaging system (Biorad, Hercules, CA, USA).

### 4.6. Statistics

Values are expressed as the mean ± standard error of the mean (SEM) from at indicated replicate samples in each experimental group; experiments were replicated to ensure consistency. Statistical significance was determined using Student’s *t*-test. Values as * was considered to be statistically significant if their *P* values were 0.05 > *P*. Signal-to-background ratio (S/B), signal-to-noise ratio (S/N), and Z’-factor were calculated by formula as described in our previous report [20]. Besides that, general criterion sufficient to do HTS was validated according to previous report [23]. In order to calculate EC_50_ value in a concentration-dependent data, we fitted the data to Logistic curve using optimized curve fit algorithm as shown in http://docs.scipy.org/doc/scipy/referenece/generated/scipy.optimize.curve_fit.html.

## Figures and Tables

**Table 1:** Qualities of independent experiments.

	Clone No.	S/B	S/N	Z’-Factor	Conc. of IL-18	Number of Sample
384 well	1	157.6	2680.8	0.842	300 ng/mL	*n* = 3
	2	94.0	1006.7	0.910	300 ng/mL	*n* = 4
	1	171.6	2921.1	0.968	100 ng/mL	*n* = 3
	2	26.5	442.6	0.745	5 ng/mL	*n* = 4

## Supplementary Materials

The following are available online at https://www.mdpi.com/1660-3397/18/1/60/s1, Sequence S1: human IL18R1 mRNA, Sequence S2: human IL18Rap mRNA, Sequence S3: Primer for pEB-IL18Rap-P2A-IL18R1-Puro, Figure S1: Supplementary figures.
