# Supplementary material for: Novel Reporter System Monitoring IL-18 Specific Signaling Can Be Applied to High-Throughput Screening
Source: Mar Drugs. 2020 Jan 17;18(1):60. doi: 10.3390/md18010060 (PMC7024245; doi:10.3390/md18010060)
Supplement: Supplementary file 1 [file marinedrugs-18-00060-s001.zip › Supplementary information/Primer for pEB-IL18Rap-P2A-IL18R1-Puro.rtf]

pEB-IL18Rap-P2A-IL18R1-Puroprimer 1AAGTCGACATGCTCTGTTTGGGCTGGATATTTCTTTGGCTTGTTprimer 2AGGTCCAGGGTTCTCCTCCACGTCTCCAGCCTGCTTCAGCAGGCTGAAGTTAGTAGCTCCGCTTCCCCATTCCTTAGGCTGGGAGCTCCTCprimer 3GGAAGCGGAGCTACTAACTTCAGCCTGCTGAAGCAGGCTGGAGACGTGGAGGAGAACCCTGGACCTATGAATTGTAGAGAATTACCCTTGACCCTTTGGGTGprimer 4TTGCGGCCGCTCAGTGGAGCCCTGAGCTTGTTTTCCTG
