# Supplementary material for: Novel Reporter System Monitoring IL-18 Specific Signaling Can Be Applied to High-Throughput Screening
Source: Mar Drugs. 2020 Jan 17;18(1):60. doi: 10.3390/md18010060 (PMC7024245; doi:10.3390/md18010060)
Supplement: Supplementary file 1 [file marinedrugs-18-00060-s001.zip › Supplementary information/Supplementary figures.pptx]

## Slide 1
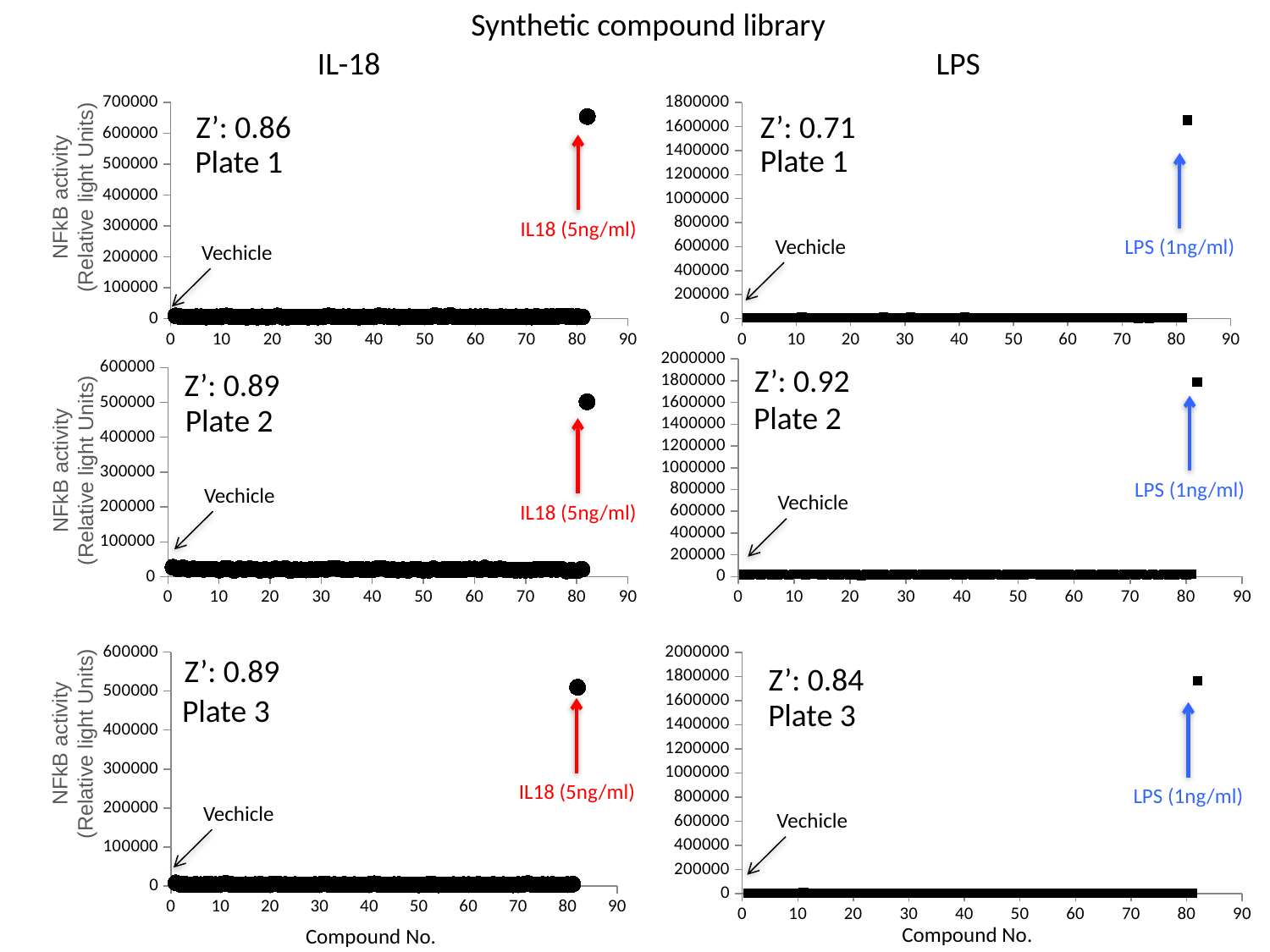

Synthetic compound library
IL-18
LPS
### Chart
| Category | |
|---|---|
### Chart
| Category | |
|---|---|Z’: 0.86
Z’: 0.71
Plate 1
Plate 1
NFkB activity
(Relative light Units)
IL18 (5ng/ml)
Vechicle
LPS (1ng/ml)
Vechicle
### Chart
| Category | |
|---|---|
### Chart
| Category | |
|---|---|Z’: 0.92
Z’: 0.89
Plate 2
Plate 2
NFkB activity
(Relative light Units)
LPS (1ng/ml)
Vechicle
Vechicle
IL18 (5ng/ml)
### Chart
| Category | |
|---|---|
### Chart
| Category | |
|---|---|Z’: 0.89
Z’: 0.84
Plate 3
Plate 3
NFkB activity
(Relative light Units)
IL18 (5ng/ml)
LPS (1ng/ml)
Vechicle
Vechicle
Compound No.
Compound No.
